# Undergraduate students’ attitudes to COVID-19 during the lockdown period: Hierarchy of psychological factors

**DOI:** 10.1192/j.eurpsy.2021.711

**Published:** 2021-08-13

**Authors:** E. Nikolaev

**Affiliations:** Social And Clinical Psychology, Ulianov Chuvash State University, Chebokasry, Russian Federation

**Keywords:** psychological factors, undergraduate students, attitudes, COVID-19

## Abstract

**Introduction:**

The effect of COVID-19 on different age groups is not the same. It is of great interest to see how specifically students, who are regarded as a less susceptible group, relate to COVID-19 during the period of government imposed lockdown.

**Objectives:**

To determine the factorial structure of the revealed university students’ attitudes to COVID-19 during the period of lockdown and distance learning.

**Methods:**

We questioned online 127 male and 200 female Russian universities students during their distance learning. We used a 17-point Attitude towards COVID-19 Questionnaire based on the results of the half-structured interview with the students. We subjected the received data to a factor analysis.

**Results:**

With the principal components method, we obtained a five-factor structure of the questionnaire under study with the total variance of 65.2%. According to the content of the questions, we defined these factors in the following way: factor of COVID-19 danger to the society (with variance – 20.2%); factor of ruined personal life plans (14.0%); factor of COVID-19 threat to personal health and life (13.9%); factor of disbelief in COVID-19 dangers (9.1%) and factor of expecting new pandemics (8.0%). Here is the hierarchy of the mean numbers of students who had maximal points in each of the factors mentioned: 66.6%; 59.3%; 24.4%; 23.9% and 23.2%.

**Conclusions:**

The students’ attitudes to COVID-19 depended on different tendencies during the lockdown period. The prevailing perception of COVID-19 as a real threat to health and life went together with the undervaluation of its significance and a shift to everyday life issues.

